# Induction of proteasomal activity in mammalian cells by lifespan-extending tRNA synthetase inhibitors

**DOI:** 10.1007/s11357-023-00938-8

**Published:** 2023-09-25

**Authors:** Blaise L. Mariner, Antonio S. Rodriguez, Olivia C. Heath, Mark A. McCormick

**Affiliations:** 1 Department of Biochemistry and Molecular Biology, School of Medicine, University of New Mexico Health Sciences Center, Albuquerque, NM 87131 USA; 2 Center for Biomedical Engineering, University of New Mexico, Albuquerque, NM 87131 USA; 3 Autophagy, Inflammation and Metabolism Center of Biomedical Research Excellence, Albuquerque, NM 87131 USA

**Keywords:** Gcn4, ATF-4, ATF4, tRNA synthetase, Ubiquitin proteasome system

## Abstract

We have recently shown that multiple tRNA synthetase inhibitors can greatly increase lifespan in multiple models by acting through the conserved transcription factor ATF4. Here, we show that these compounds, and several others of the same class, can greatly upregulate mammalian ATF4 in cells in vitro, in a dose dependent manner. Further, RNASeq analysis of these cells pointed toward changes in protein turnover. In subsequent experiments here we show that multiple tRNA synthetase inhibitors can greatly upregulate activity of the ubiquitin proteasome system (UPS) in cells in an ATF4-dependent manner. The UPS plays an important role in the turnover of many damaged or dysfunctional proteins in an organism. Increasing UPS activity has been shown to enhance the survival of Huntington’s disease cell models, but there are few known pharmacological enhancers of the UPS. Additionally, we see separate ATF4 dependent upregulation of macroautophagy upon treatment with tRNA synthetase inhibitors. Protein degradation is an essential cellular process linked to many important human diseases of aging such as Alzheimer’s disease and Huntington’s disease. These drugs’ ability to enhance proteostasis more broadly could have wide-ranging implications in the treatment of important age-related neurodegenerative diseases.

**Supplementary Information:**

The online version contains supplementary material available at 10.1007/s11357-023-00938-8.

## Introduction

The ubiquitin proteasome system (UPS) is essential for the turnover of many polypeptides in eukaryotes, but is especially responsible for the turnover of the dysfunctional, mutated, misfolded, or damaged proteins [1]. The UPS has been hypothesized as a high-impact target to treat many diseases such as Huntington’s disease, Alzheimer’s disease, and Parkinson’s disease, among others [2–7]. Increased UPS activity has also been hypothesized to improve an organism’s ability to maintain protein homeostasis, a hallmark of aging [3, 4, 8–15]. Drugs that activate or enhance proteasome activity are rare, especially in comparison to proteasome inhibitors [6].

Activating transcription factor 4 (ATF4) is a conserved transcription factor of recent interest in its wide variety of diseases such as neurodegeneration, diabetes, cancer, and skeletal muscle aging [16–27]. The *Atf4* orthologs *GCN4* / *atf-4* have been linked to increased lifespan in both the budding yeast *S. cerevisiae* and the nematode *C. elegans* [18, 21, 28–31]. Further, long-lived mice have been shown to have elevated levels of ATF4 [32, 33].

There are four kinases in vertebrates that are known to upregulate ATF4 translation through the integrated stress response (ISR), which can be activated in response to endoplasmic reticulum stress, amino acid deprivation, the presence of double stranded RNA, and, in erythroid cells, heme deficiency [16]. These four kinases phosphorylate the alpha subunit of eukaryotic initiation factor 2 (eIF2α), an essential protein in the formation of the translation pre-initiation complex in eukaryotes. This eIF2α phosphorylation results in the delay in translational re-initiation and subsequent ATF4 translation [16, 34]. General control non-derepressible kinase 2 (GCN2) is an eIF2α kinase and the activator of the amino acid response leg of the ISR [35]. GCN2 autophosphorylates and activates upon the kinase’s binding to uncharged tRNA [20, 34, 36, 37].

There are two important modes of protein degradation in the cell. First, autophagy is a degradation process in which cellular components, such as proteins and organelles, are delivered to the degradative organelles for breakdown and re-purposing of macromolecules, like amino acids [38]. There are many different flavors of autophagy, such as, but not limited to, mitophagy, selective autophagy, and ribophagy, all of which are only starting to be understood especially in the context of aging [39–43]. Macroautophagy, hereafter ‘autophagy’, is the most widely-studied, and has been established as an important biological process in many long-lived organisms [28, 40, 44–48]. Autophagy genes and their role in aging and disease phenotypes are widely studied and have been recently reviewed [49]. ATF4 is known to aid in the induction of autophagy through its role as a transcription factor [50–57]. Second, the UPS degrades “tagged” or poly-ubiquitinated proteins [1]. In contrast, autophagy is a form of bulk component recycling, while the proteasome turns over proteins on a more individual level, resulting in the proteasome’s tight connection to the regulation of many cellular responses [1]. Increased proteasomal capacity has been found to be responsible for some extremely long-lived phenotypes in yeast, worms and flies, indicating that further research exploring this process in mammals is of great interest to the aging field [3, 4, 8–10, 18]. Altogether, the promotion of these two processes to increase protein degradation has been shown to increase health in many models, underscoring the importance of an organism’s ability to maintain protein homeostasis in healthy aging [3, 5, 40, 58–63].

Although there is great interest in the treatment potential of UPS activators, there are few known pharmacological agents that can do so [5]. Here, we show that seven different tRNA synthetase inhibitors can dramatically induce proteasome activity in an *Atf4*-dependent manner in mammalian cells in vitro*.* We also show that these same drugs can upregulate proteasomal activity at the same doses, as well as macroautophagy, suggesting these drugs’ potential to treat important diseases of aging characterized by protein aggregation in vivo. As we have recently shown that some of these same compounds dramatically increase healthy wild-type lifespan in multiple model organisms, this also leaves open the possibility that these potential treatments for known and significant diseases of aging might also act directly on aging itself [27].

### Methods

#### Mouse embryonic fibroblast cell culturing and ATF4 KO cell line generation

Wild type and GCN2 KO mouse embryonic fibroblast (MEFs) were purchased from American Type Culture Collection global biological resource center. The ATF4 KO cell line was made in house using utilizing optimized guide RNAs optimized from the available CRISPR/Cas9 genome editing tools with Integrated DNA Technologies targeting the *Atf4* gene. Transfection of the assembled Cas9 complex utilized the NEON electroporation system by ThermoFisher and fluorescence-activated cell sorting with the sy3200 Cell Sorter from Sony Biotechnology in collaborations with the UNM Comprehensive Cancer Center Support Grant NCI P30CA118100 and the UNMHSC Flow Cytometry shared resource (Supplementary Fig. 2). All cell culture procedures were done in a BSL-2 laminar flow cabinet with cells of a low progeny. All sub-culturing procedures utilized guidance from peer-published resources, without Penicillin–Streptomycin [64, 65]. All plasmid transfections utilized Viafect (Promega) following the manufacturer instructions. Halofuginone was purchased from Ambeed [CAS No. 64924–67-0]. Borrelidin was purchased from BioViotica (BVT-0098). Thapsigargin was purchased from AdipoGen (AG-CN2-0003). Bafilomycin A1 was purchased from Sigma-Aldrich (19–148). LysRS-IN-2 was purchased from GLPBio (GC65058). REP3123 and REP8839 were purchased from Axon Medchem (1704, 1705). Mupirocin was purchased from BOC Sciences (B0084-056590). MG-132 was purchased from Sigma-Aldrich (M7449). Tavaborole was purchased from Cayman Chemical (23101).

### Fluorescence and luciferase assays in MEFs

ATF4 translation experiments utilized a fluorescent translational reporter for ATF4:ATF4 5:5’ATF4.GFP was a gift from David Ron (Addgene plasmid # 21852; http://n2t.net/addgene:21852; RRID:Addgene_21852) [36]. ATF4 translation over time was measured with the BioTek Synergy HTX Multi-Mode Microplate Reader shared resource in the UNMHSC Autophagy, Inflammation, and Metabolism Center for Biomedical Research Excellence (AIM) core. The AIM core is supported by NIH grant P20GM121176 from NIGMS. All luciferase assays utilized a Victor NIVO multimode plate reader. ATF4 downstream activity was measured using the pGL4[luc2P/ATF4-RE/Hygro] Vector, which was purchased from Promega utilizing a previously well-understood ATF4 binding element [66, 67]. Proteasomal assays utilized (1) Promega’s Proteasome-Glo Caspase-like Cell-Based Assay (G8660) and (2) Abcam’s Proteasomal Activity Kit (ab107921) according to the manufacturers’ procedures respectively [68, 69]. Protein aggregation assays utilized Enzo Life Sciences’ PROTEOSTAT® (51023) according to the manufacturers’ protocols.

All LIVE/DEAD, autophagy, and protein synthesis assays were quantified by the Cellinsight CX7 high-content screening platform by ThermoFisher in the UNMHSC AIM core. LIVE/DEAD assay utilized the ATT Bioquest Live or Dead Cell Viability Assay Kit (Cat. No. 22789). Autophagy assays utilized two autophagic flux reporters. pMRX-IP-GFP-LC3-RFP-LC3ΔG was a gift from Noboru Mizushima (Addgene plasmid # 84572; http://n2t.net/addgene:84572; RRID:Addgene_84572) and ptfLC3 was a gift from Tamotsu Yoshimori (Addgene plasmid # 21074; http://n2t.net/addgene:21074; RRID:Addgene_21074) [70, 71]. Widefield microscopy to supplement the findings utilized the UNMCCC Fluorescence Microscopy and Cell Imaging Shared Resource which is supported by University of New Mexico Comprehensive Cancer Center Support Grant NCI P30CA118100. Protein synthesis assays were conducted with the Click-iT™ HPG Alexa Fluor™ 488 Protein Synthesis Assay Kit (ThermoFisher Cat. No. C10428) following the manufacturer’s protocol, where fluorescence surrounding the cell nucleus was quantified in a high-throughput manner, representing the rate of protein synthesis for each cell.

### MEF RNA-seq and analysis

RNA was extracted from ~ 70% confluent MEFs after 7 h of condition exposure in accordance with the block design in 60-mm cell culture dishes. DMSO of 1% v/v was used for the controls and 600 nM borrelidin delivered in DMSO at congruently 1% v/v was used in accordance with the high downstream ATF4 transcriptional activity evidenced from the ATF4 downstream luciferase assays (Fig. 2E, 2F). RNA was extracted using Zymo’s RNA extraction kit according to the manufacturer’s protocols. A total of 48 samples were sent to GeneWiz and all except two passed RNA quality control tests, resulting in *n* ≥ 11 for each condition. After read quality control and fastp processing, fastq files were aligned and quantified utilizing HISAT2 and feature counts [72–75]. Data analysis and differential expression utilized R and the libraries limma, for differential expression analysis and creation of the linear model, and DESEQ2, for data visualization and differential expression analysis [76, 77].

### Western blotting analysis of MEF proteins

Cell protein extraction and Western blot analyses were done using standard procedures. Briefly, protein samples were extracted using RIPA Lysis and Extraction Buffer (Genesee Sci. Cat. No. 18–415) using manufacturer’s protocol followed by suspension in Laemmli Sample Buffer (Bio-rad Cat. No. #1610737EDU) and heating to 98 °C for 5 min to denature the proteins. Protein was quantified and loaded equally into electrophoresis gels using Pierce™ BCA Protein Assay Kit (ThermoFisher Cat. No. 23225). The protein samples were loaded into Novex™ 4 to 20% or 10% Tris–Glycine Plus, 1.0 mm, Midi Protein Gels (Invitrogen Cat. No. WXP42012BOXA) in Tri/Glycine/SDS buffer. After electrophoresis, the gel was transferred to an Immun-Blot® PVDF Membrane (Bio-rad Cat. No. #1620175) in Tris/glycine transfer buffer with 10% methanol. Membranes were blocked in 5% dried milk in TBST with tween. The membranes were immunoblotted with the following primary antibodies: LC3 (Sigma, Cat. No. L8918), ATF4 (Proteintech Cat. No. 10835–1-AP), eIF2α (ThermoFisher Cat. No. PA5-41916), Phopho-eIF2α (ThermoFisher Cat. No. 44-728G). 10 μg/well of protein was loaded for ATF4 and LC3 western blots while 20 μg/well was loaded for eIF2α and Phospho-eIF2α blots. The secondary antibody used was an appropriate horseradish peroxidase-conjugated antibody, in which the membranes were incubated in for 1 h at room temperature. The antibody-antigen complex was visualized by a Clarity™ Western ECL Substrate (Bio-rad Cat. No. 170–5061). The intensities of the bands were quantified using the Gel Imager program, by normalizing the band intensity of proteins of interest to the lane’s total protein quantified by Ponceau [78].

### Statistical analysis

Statistical significance of ATF4 fluorescent and luciferase assays was calculated using the Dunnett’s multiple comparison procedure versus the vehicle. Statistical significance for all autophagy fluorescence assays were calculated using the Dunnett’s multiple comparison procedure versus the vehicle. Statistical significance of all western blots were calculated using a one-way ANOVA. All data displaying the changes in ATF4 levels, translation, or transcriptional reporter activity are normalized to the vehicle control, representing fold change (FC) with error bars representing the standard error of the normalized fold change mean. Unless specified otherwise, all error bars are standard error of the mean. Any noted statistical significance is reported in the figure legend. Significantly differentially expressed genes were found from the limma R package, using the Kenward-Roger approximation for linear models. Over-represented ontologies were calculated using both Panther and ClueGo using Benjamini–Hochberg multiple testing correction for False Discovery Rate calculation. All caspase-like and chemotrypsin-like assay *p*-values were calculated utilizing a one-way ANOVA. All caspase-like and chemotrypsin-like assay data presented is presented as fold change (FC) in relation to the vehicle control. All statistics and display of data was done with R programming.

## Results

### Multiple tRNA synthetase inhibitors can increase ATF4 in mammalian cells

**Fig. 1:**
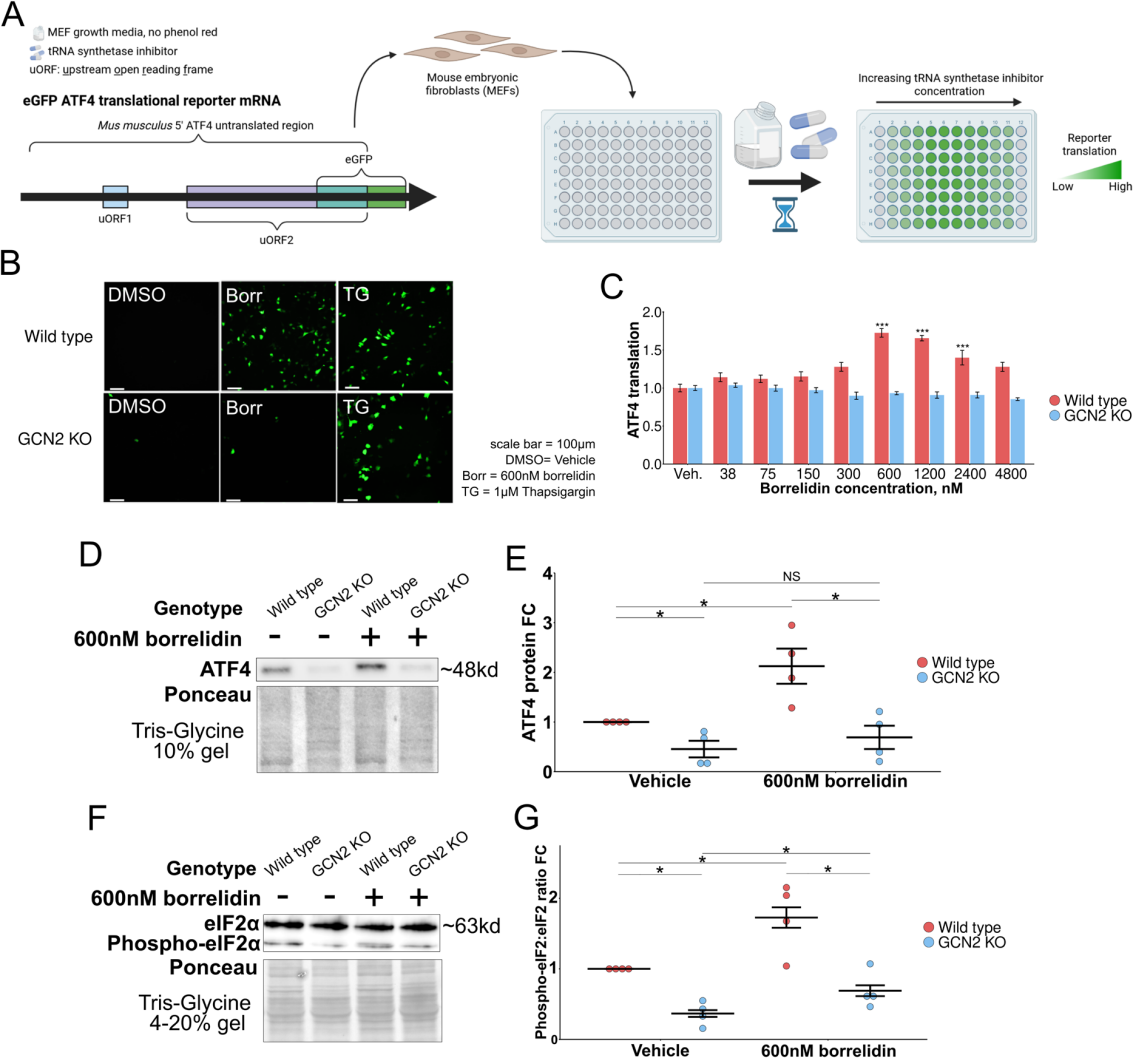
tRNA synthetase inhibitors can increase ATF4 levels in mouse embryonic fibroblasts. **A** The ATF4 eGFP translation reporter utilizes ATF4’s 5′ untranslated region upstream of the GFP start codon [37]. The study design first used the eGFP ATF4 translation reporter to find concentrations of tRNA synthetase inhibitor that upregulate ATF4 translation. **B**, **C** ATF4 translation measured in response to varying concentrations of borrelidin (*p < 0.05, **p < 0.01, ***p < 0.001; Dunnett’s multiple testing procedure). **D**, **E** ATF4 protein levels in response to borrelidin, normalized to total protein level detected by ponceau stain. **F**, **G** Phospho-eIF2 and eIF2 protein measured ratio in response to borrelidin, normalized to total protein level detected by ponceau stain (*p < 0.05, **p < 0.01, ***p < 0.001; one-way ANOVA). eGFP = enhanced green fluorescent protein, Veh. = Vehicle, FC = fold change

ATF4 is regulated translationally by two upstream open reading frames (uORFs) in its 5′ untranslated region, and a delay in translation re-initiation results in increased ATF4 translation [36, 37]. We first measured ATF4 translation in response to tRNA synthetase inhibitors using an eGFP-ATF4 translational reporter containing the 5′ untranslated region of ATF4 fused to eGFP in place of ATF4 in mouse embryonic fibroblasts (MEFs) (Fig. 1A) [36]. We first sought to ask if borrelidin, a threonyl tRNA synthetase inhibitor, could increase ATF4 translation through the GCN2-mediated amino acid response leg of the integrated stress response [79, 80]. To find the therapeutic window in culture, we first assessed cell viability in an increasing doses of drug and found that 24 h of 2 mM borrelidin treatment in media almost completely killed the cells (Supplemental Fig. 1A). From there, we dialed in the treatment incubation time over a dose range of 0 to 4.8 mM and found that 7 h was consistently sufficient to upregulate ATF4 translation at doses from 150 to 2400 nM (Supplemental Fig. 1B). After that, we found that this ATF4 upregulation was dependent on *Gcn2*, utilizing thapsigargin (TG), an inducer of the ISR through the PKR-like endoplasmic reticulum kinase, as a positive control (Fig. 1B, C, Supplemental Fig. 1B). We confirmed this ATF4 upregulation with western blots (Fig. 1D, E, Supplemental Fig. 1C, 1D). To further test if tRNA synthetase inhibitors are acting through the AAR, we assessed the ratio of phosphorylated-eIF2α to eIF2α with western blots (Fig. 1F, G, Supplemental Fig. 1E). We found both wild type and GCN2 KO MEFs had a significant rise in eIF2α phosphorylation levels in relation to its vehicle counterpart, however the GCN2 KO cells, however, they were not significantly higher than the wild type vehicle condition. Together, these data suggest that borrelidin acts through the ISR, specifically through the phosphorylation of eIF2α by the GCN2 uncharged tRNA sensor, in order to increase ATF4 translation.

**Fig. 2:**
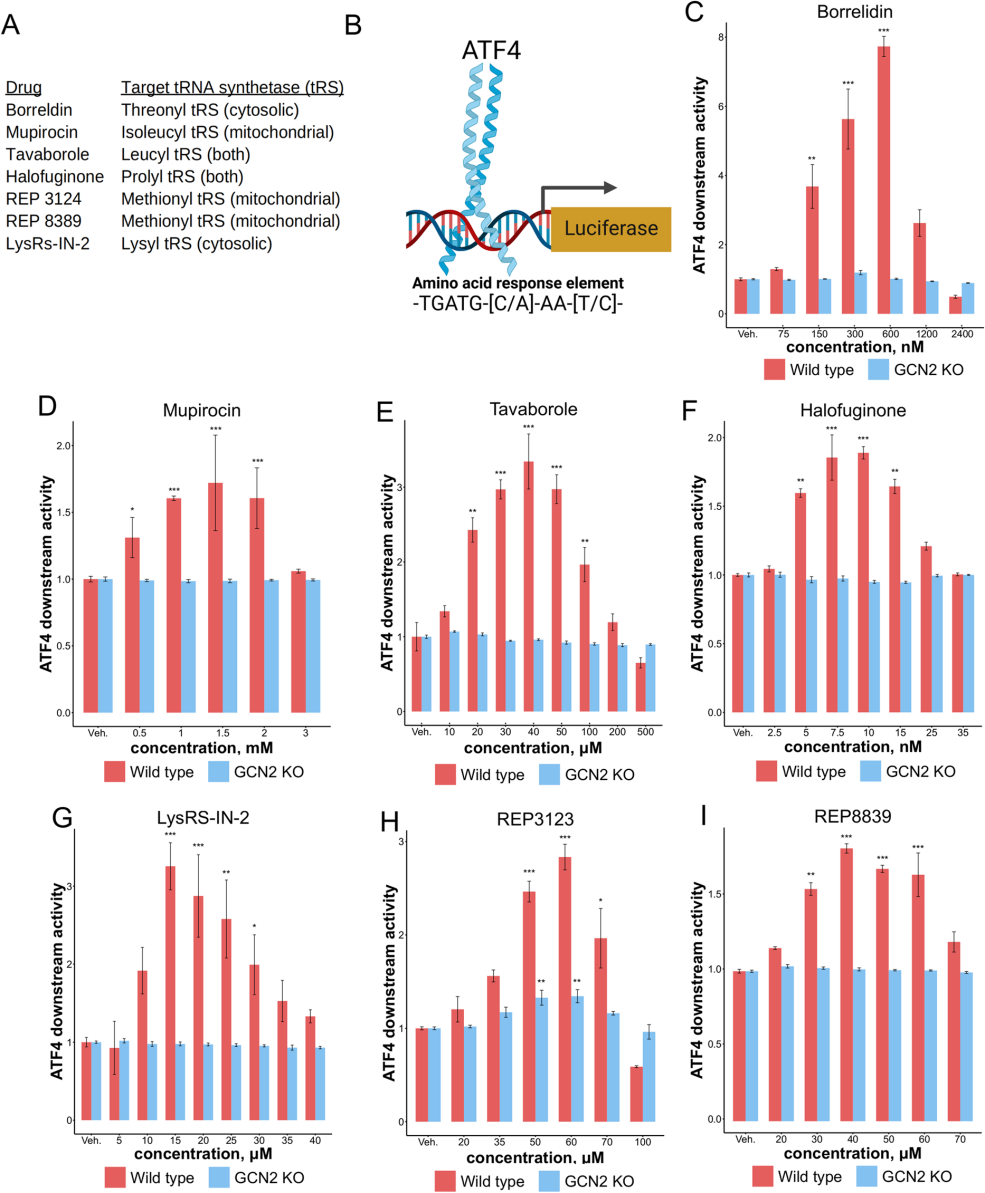
tRNA synthetase inhibitors upregulate ATF4 activity through *Gcn2*. **A** Drugs used in this study and their associated targets. **B** The amino acid response element measures ATF4 downstream activity. **C**–**I** Various tRNA synthetase inhibitors can increase ATF4 downstream activity (*p < 0.05, **p < 0.01, ***p < 0.001; Dunnett’s multiple testing procedure)

We next tested six additional inhibitors targeting different tRNA synthetases (Fig. 2A). We found that a 7-h incubation time with these drugs was sufficient to increase ATF4 translation in wild type MEFs, similar to what we saw with borrelidin (Supplemental Fig. 1F). We next utilized an amino acid response element luciferase reporter where luciferase is only transcribed when ATF4 binds to its known consensus binding sequence (Fig. 2B) [66, 67, 81]. All of these inhibitors showed a bell-shaped response curve of ATF4 downstream activity with inhibitor dose, after 7 h of treatment (Fig. 2C–I). At lower doses, activation increases with increasing drug concentration via GCN2-mediated increased translation of ATF4. At higher doses, these drugs likely inhibit overall translation enough to offset the specific induction of ATF4. Borrelidin was the most potent ATF4 activity inducer, increasing its activity by more than sevenfold at 600 nM after 7 h of treatment (Fig. 2C). Mupirocin, despite targeting the mitochondrial isoleucyl tRNA synthetase, was similarly found to increase ATF4 downstream activity and translation through *Gcn2* (Fig. 2D, Supplemental Fig. 1G) [82, 83]. The antifungal agent tavaborole, a leucyl tRNA synthetase inhibitor, was found to increase ATF4 translation and downstream activity through *Gcn2* (Fig. 2E, Supplemental Fig. 1G) [82]. Previous work utilizing halofuginone, a prolyl tRNA synthetase inhibitor, has been shown to increase ATF4 levels in vitro, and our studies confirm this (Fig. 2F, Supplemental Fig. 1D) [54]. Lys-RS-IN-2, a lysyl tRNA synthetase inhibitor, was also found to increase ATF4 activity dependent on *Gcn2* (Fig. 2G, Supplemental Fig. 1G) [84]. REP3123 and REP8839, two methionine tRNA synthetase inhibitors known for their potent affinity for the prokaryotic tRNA synthetase enzyme, were similarly found to increase ATF4 activity (Fig. 2H, I) [85, 86]. For all inhibitors except for REP3123, induction of ATF4 downstream activity was completely dependent on *Gcn2*. We reasoned that methionine tRNA synthetase inhibitors may be able to induce ATF4 independently of *Gcn2*, as charged methioninyl-tRNA is a component of the translation pre-initiation complex and thus translation initiation may be delayed when methionine tRNA synthetase activity is impacted [34, 87]. Taken together, these data suggest that ATF4 translation and downstream activity can be upregulated in response to multiple inhibitors of different tRNA synthetases in mammalian cells.

### ATF4 upregulation causes the differential expression of genes associated with protein turnover

**Fig. 3:**
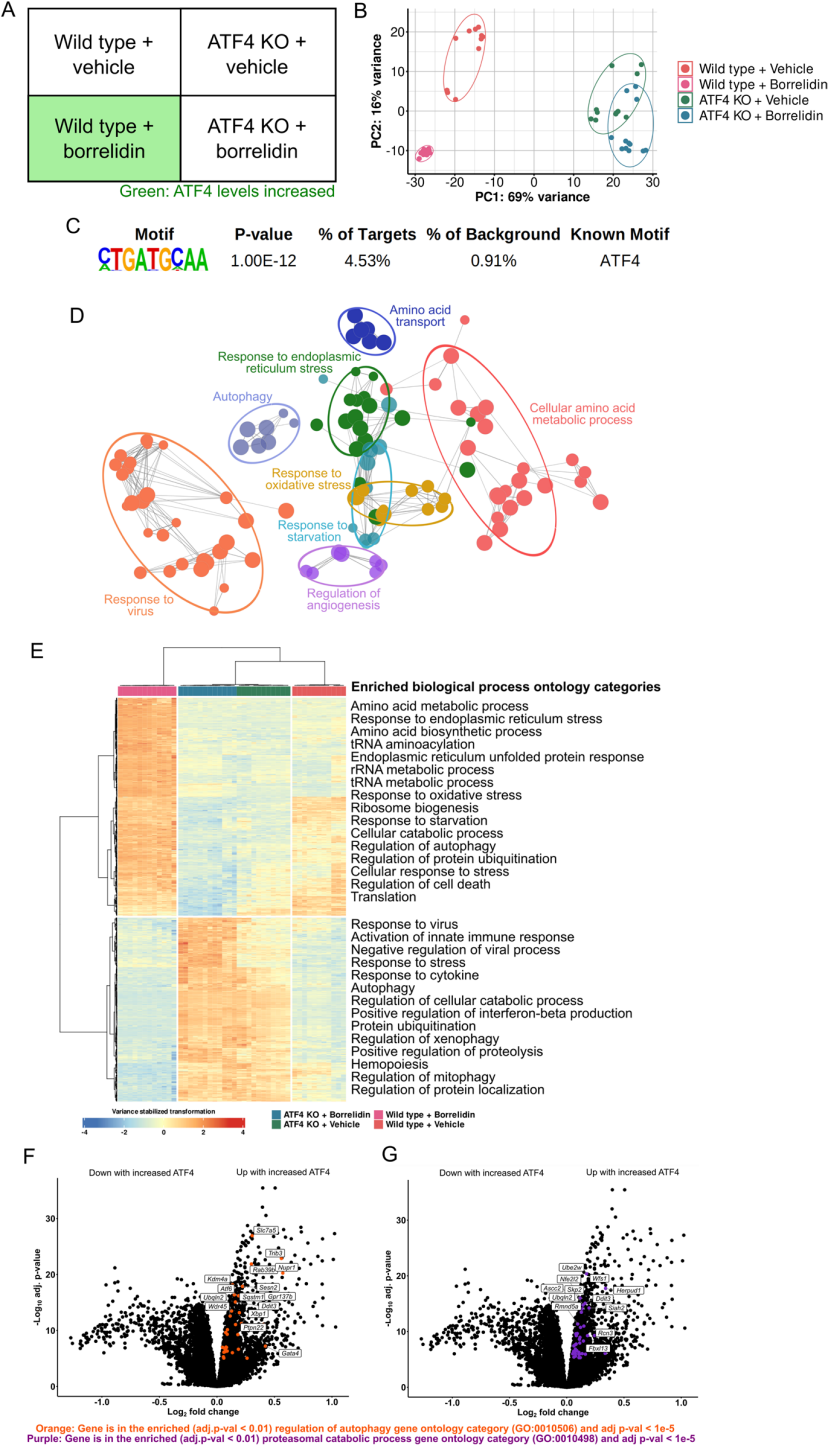
ATF4 causes the differential expression of genes involved with protein turnover. **A** RNASeqblock design to control for borrelidin treatment and *Atf4* knockout sequencing. **B** Principal component analysis of the 46 samples sequenced. **C** HOMER motif enrichment from the differentially expressed genes associated with ATF4 (adj. *p*-value < 1e-10). D) ClueGo enriched biological process ontology categories (adj. *p*-value < 0.01) of differentially expressed genes (adj. *p*-value < 1e-10) from the linear model result (Design =  ~ ATF4 + Condition). **E** Heatmap and hierarchical clustering of linear model results (*p* adj < 1e-10). Biological process gene ontology enrichment utilized Panther’s ontology resource. **F**, **G** Volcano plots of genes differentially expressed from linear model. Both regulation of autophagy (in orange) and proteasomal protein catabolic process (in purple) were found to be enriched in the genes upregulated (p adj < 1e-10) with ATF4 (Panther’s ontology resource, adj. *p*-value < 0.01). “Up with increased ATF4” and “Down with increased ATF4” are labeled on either side of the volcano plots presented in association with the limma linear model fit to ATF4 activity given by the linear model study design (Design =  ~ ATF4 + Genotype)

After we identified highly inducing doses of tRNA synthetase inhibitors in MEFs, we sought to analyze the ATF4-dependent changes in the cell’s transcriptome. We knocked out *Atf4* using an Integrated DNA Technologies-adapted CRISPR-Cas9 protocol (Supplementary Fig. 2) and we designed an RNA-sequencing experiment to analyze the ATF4 transcriptional targets under high activity using linear model analysis of a block design with high biological replicates (*n* ≥ 11 per treatment) (Fig. 3A) [76, 88]. The block design features the ability to simultaneously control for the two variables: (1) tRNA synthetase inhibitor treatment and (2) ATF4 activity levels. We chose to conduct the RNASeq with borrelidin as it was the most potent inducer of the ATF4 transcriptional response. Principal component analysis of the 46 samples revealed that principal component 1 was able to separate the samples by their genotype while principal component 2 separated based on the treatment of drug (Fig. 3B). Principal component 2 separated the wild type samples based on their drug treatment much better than it did the ATF4 KO samples, suggesting that borrelidin impacted the two different cell lines differently, and in line with our expectation that many of the transcriptional changes upon borrelidin treatment might depend on increased ATF4 translation.

After alignment of reads and quantification, genes with changed mRNA levels associated with ATF4 activity were pulled out based on the linear model fit to increased ATF4 levels (Interactive volcano plot of results provided in File 1) [76]. Figure 3 C–F present the findings of this linear model design (Design =  ~ ATF4 + Genotype), which upon fitting, results in the log fold change and adjusted *p*-values of transcripts in association with the documented ATF4 activity from Fig. 2. Using HOMER motif analysis, we confirmed that the immediately upstream presumptive promoter sequences of genes whose transcript levels changed with ATF4 activity (*p*_adj_ < 1e-5) showed enrichment of the known ATF4 amino acid response binding element [A/G]-TT-[G/T]-CATCA (*p* < 1e-12) (Fig. 3C) [66, 67, 89]. The enriched biological process ontology categories from the genes differentially expressed from the linear model fit (*p*_adj_ < 1e-10) were plotted on an edge-node graph utilizing ClueGo (Fig. 3D) [90].

We next used hierarchical clustering of the genes found to be significantly (*p*_adj_ < 1e-10) associated with linear model fit (Fig. 3E). Encouragingly, the samples (columns) clustered cleanly in alignment with their genotype and drug treatment. This clustering further revealed that genes associated with autophagy, protein catabolism, translation, and other stress responsive genes are differentially expressed downstream of ATF4. We also analyzed the enriched biological process gene ontology categories of the genes upregulated in conditions of high ATF4 activity, and found many processes involved in protein degradation were over-represented (regulation of autophagy (GO:0010506), proteasomal protein catabolic process (GO:0010498), ERAD pathway (GO:0036503)) (Fig. 3F, G, interactive volcano plot included in File 1). Further, these data present differentially expressed genes known to impact protein synthesis, by the amino acid biosynthesis ontology categories shown to be enriched here, in alignment with what is already known about *Atf4* and its orthologs [16, 91]. Altogether, these data indicate that ATF4 may be a key regulator of protein turnover, changing the expression of genes involved in protein synthesis and protein degradation.

Many novel genes were found to be significantly differentially expressed with ATF4 activity, including *Wipi2*, a gene essential in the LC3 lipidation step of autophagy, which was upregulated with high ATF4 activity [92, 93]. Quantitative PCR of autophagy genes *Atg2a*, *Atg7*, *Atp13a2*, and *Wipi2* further confirmed higher levels of the associated genes’ mRNA in borrelidin-treated wild type samples, in agreement with our RNASeq results for these genes (*p* < 0.05) (Supplemental Fig. 3A). Our RNASeq results also showed increased expression of a significant number of genes encoding tRNA synthetases when ATF4 is induced, including *Tars*, *Gars*, *Cars*, *Nars*, *Yars*, *Sars*, *Lars*, *Wars*, *Mars1*, *Vars*, and *Eprs* (*p*_adj_ < 1e-3), consistent with other studies (Supplemental Fig. 3B, 3C, 3D) [52, 94]. Conceptually, these data suggest that ATF4 increases the expression of these tRNA synthetases to correct for the scenario that the ISR was activated by the accumulation of uncharged tRNAs, potentially due to a malfunctioning tRNA synthetase.

### tRNA synthetase inhibitors and ATF4 can decrease protein synthesis

**Fig. 4:**
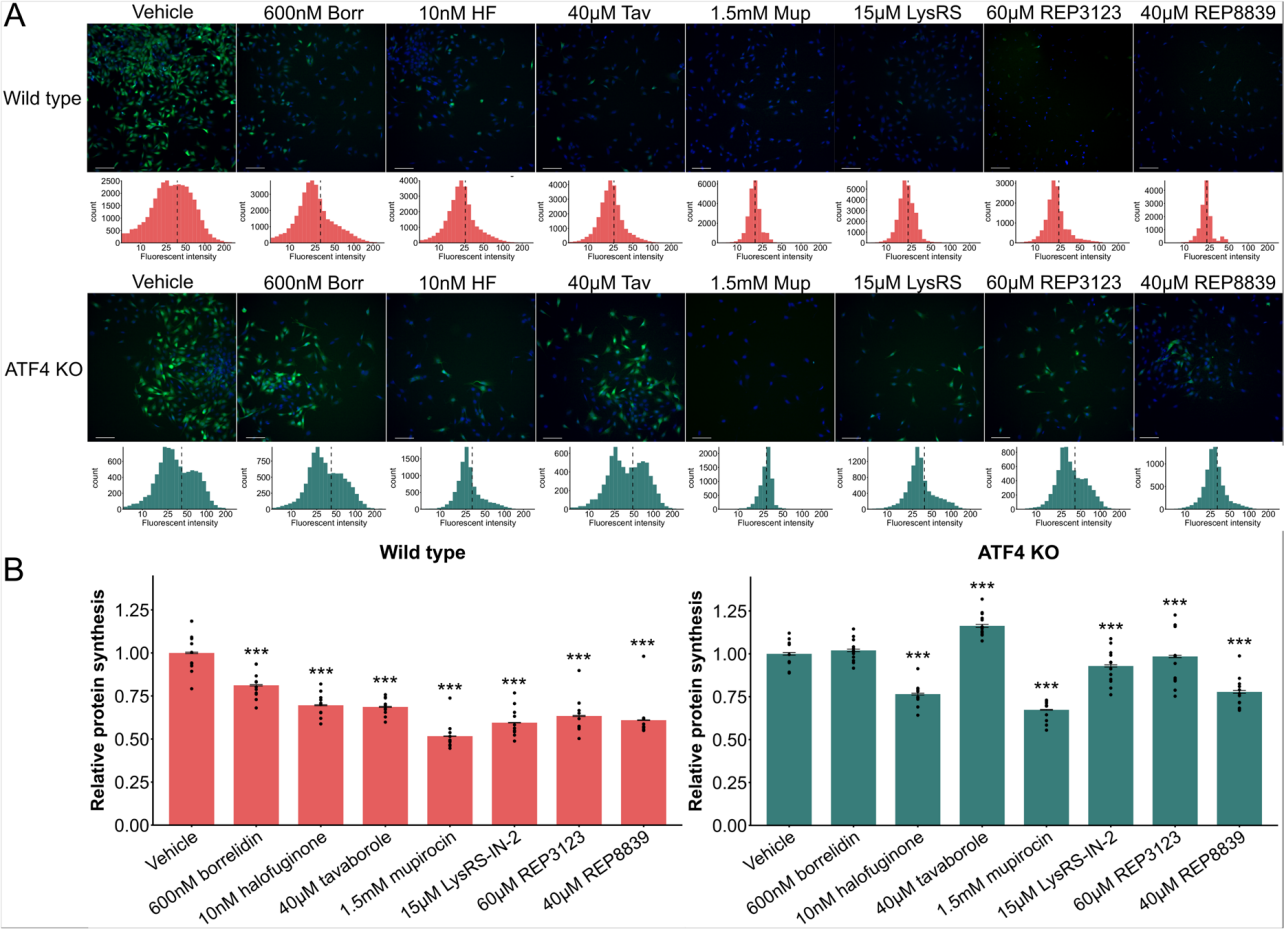
tRNA synthetase inhibitors and ATF4 can decrease protein synthesis in MEFs. **A** Representative images and histograms of green fluorescence, representing protein synthesis, of wild type and ATF4 KO MEFs treated with various tRNA synthetase inhibitors. Green: Alexa Fluor.™ 488. Blue: NuclearMask™ Blue Stain. **B** Relative protein synthesis in wild type and ATF4 KO MEFs, normalized to wild type vehicle control in all cases. Dots represent different experiments. (*p < 0.01; **p < 0.01, ***p < 0.001; Students’ *t*-test)

From the RNASeq results, there were many genes found to be differentially expressed with increased ATF4 activity having to do with translation, indicating that ATF4 impacts protein synthesis, aligning with other studies [16, 91, 95, 96]. As protein turnover mechanisms are tightly controlled by the balance between protein synthesis and degradation, we sought to understand the impact of our drugs and ATF4 on protein synthesis. We found that wild type cells treated with every different tRNA synthetase inhibitor at doses that increase ATF4 levels had significantly reduced translation (Fig. 4A). We next assessed the impact of tRNA synthetase inhibitors on ATF4 KO cells and found that 5/7 tRNA synthetase inhibitors had reduced translation, while borrelidin and tavaborole either had no significant change, or increased protein synthesis. Consistently, wild type cells have lower protein synthesis than ATF4 KO cells when treated with the same dosage of drug, implicating ATF4 as a reducer of protein synthesis, in agreement with other studies [16, 91, 95, 96]. However, cells treated with halofuginone, mupirocin, LysRS-IN-2, REP3123, and REP8839 still had significantly reduced protein synthesis in comparison to their vehicle counterpart in both wild type and ATF4 KO cells (Fig. 4B).

### tRNA synthetase inhibitors can upregulate protein degradation in an Atf4 dependent manner

**Fig. 5:**
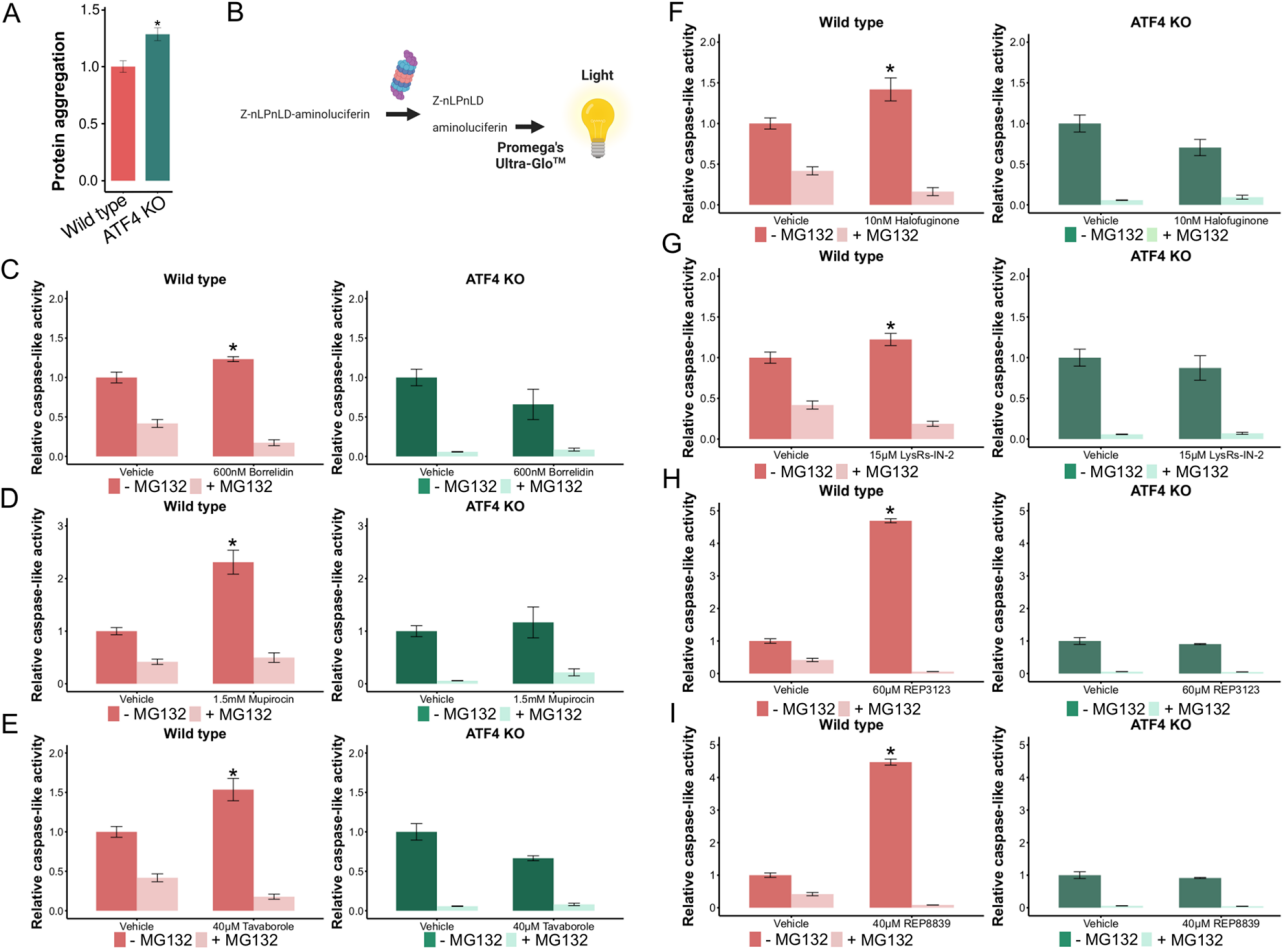
tRNA synthetase inhibitors increase caspase-like activity through *Atf4*. **A** Peptide and protein aggregation of wild type and ATF4 KO MEFs. Protein aggregation assessed using PROTEOSTAT ® divided by the total protein in the sample, normalized to wild type. **B** Promega’s assay schema to measure caspase-like activity of the proteasome. C–I Caspase-like activity increases in response to different tRNA synthetase inhibitors at ATF4-inducing concentrations (*p < 0.05, **p < 0.01, ***p < 0.001; one-way ANOVA)

Protein turnover in an organism is balanced by both protein synthesis and protein degradation. This knowledge, along with the results from the RNASeq indicating a change in autophagy and proteasomal activity, led us to next assess peptide and protein aggregation in cell lines with and without *Atf4* (Fig. 5A). Intriguingly, the untreated ATF4 KO cell line had significantly more aggregation than the untreated wild type cell line, which could be due to impaired unfolded protein response in ATF4 KO cells [97]. ATF4 is also among the individual proteins degraded by the UPS, suggesting the possible existence of a negative feedback loop [98, 99]. To further investigate ATF4’s influence on protein degradation, we assessed the proteasome’s caspase-like activity (Fig. 5B). We found that borrelidin is able to significantly increase proteasomal caspase-like activity in wild type MEFs (Fig. 5C). However, in ATF4 KO MEFs, borrelidin did not increase proteasomal caspase-like activity, suggesting that *Atf4* is necessary for increased caspase-like proteasomal activity upon borrelidin treatment. Similar results were found in all of the tested tRNA synthetase inhibitors at concentrations that we found them to induce ATF4 activity (Fig. 5D–I). Interestingly, the mitochondrial methionine tRNA synthetase inhibitors, REP3124 and REP8839, induced proteasomal caspase-like activity the most, agreeing with previous work on responses to direct methionine restriction [100]. We also assessed the proteasome’s chemotrypsin-like activity, and found chymotrypsin-like proteasomal activity to be induced in an *Atf4*-dependent manner by all 7 inhibitors as well, mirroring our findings with caspase-like proteasomal activity (Supplemental Fig. 4A-4H). Taken together, these data show that tRNA synthetase inhibitors can increase proteasomal activity in mammalian cells in an *Atf4*-dependent manner.

As genes involved in autophagy were also identified in our RNASeq results (Fig. 3D–F), we next assessed the change in autophagy in response to tRNA synthetase inhibitors, and found that autophagic flux is upregulated upon tRNA synthetase inhibitor treatment, completely dependent on *Atf4*. We demonstrated this utilizing three different, independent autophagy assays. The first assay used an autophagic flux GFP-LC3-RFP-(LC3ΔG) fluorescent reporter, and showed that autophagic flux increases in tRNA synthetase inhibitor treated samples, dependent on *Atf4* (Supplemental Fig. 5A, 5B). We next utilized the ptfLC3 autophagic flux reporter, which again showed that autophagic flux levels rose in response to tRNA synthetase inhibitors, dependent on *Atf4* (Supplemental Fig. 5C, 5D, 5E, 5F). Finally, we quantified levels of LC3II by western blot and found that they were increased upon tRNA synthetase inhibitor treatment, again dependent on *Atf4* (Supplemental Fig. 5G, 5H). LC3II levels are linked to the formation of the autophagosome and increased autophagy [49]. These results further indicate that tRNA synthetase inhibitors can increase autophagy in an *Atf4*-dependent manner, aligning with other studies [54]. Altogether, these results show tRNA synthetase inhibitors can increase protein degradation pathways through *Atf4* in mammals, outlining *Atf4* as a key contributor to the regulation of protein turnover.

## Discussion

**Fig. 6:**
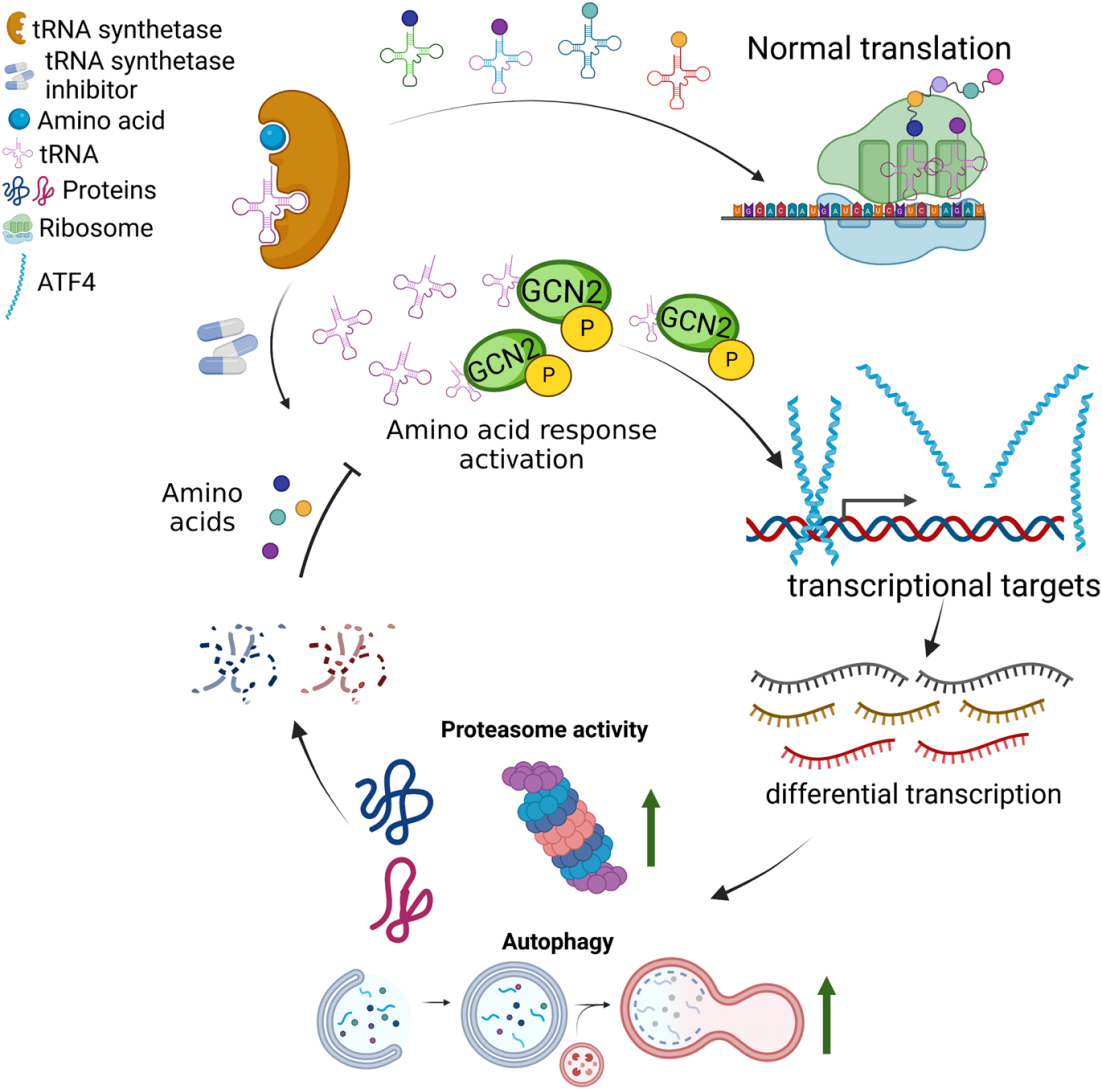
tRNA synthetase inhibitors cause the activation of the integrated stress response. Proteasome activity and autophagy play a role in the negative feedback loop of amino acid response pathway activation

Pharmacological inducers of the UPS are scarce but have the potential to treat many diseases such as Huntington’s disease, Alzheimer’s disease, and Parkinson’s disease [2, 3, 5, 58, 101, 102]. Pharmacological autophagy inducers also have the potential to play roles in protection against neurodegenerative diseases and aging [103–105]. Notably, we found multiple distinct tRNA synthetase inhibitors can dramatically upregulate the UPS in mammalian cells through *Atf4* (Fig. 6) [98, 99]. The drugs presented here, including two tRNA synthetase inhibitors that have been shown to increase lifespan in yeast and worms, have major implications for the biological activity of ATF4 and its potential impact on the aging process [27]. Further, the unfolded protein response is a pathway ending with ATF4 that is known to increase lifespan in many model organisms [97, 106–109]. This study, along with the growing literature surrounding ATF4, underscores the highly important role ATF4 potentially plays in many longlived models that may converge on improved proteostasis, itself a hallmark of aging [11–13, 110, 111].

Cells are likely to interpret uncharged tRNAs as a sign of amino acid starvation, regardless of whether the uncharged tRNA stems from the cytoplasm or mitochondria [112]. Given that GCN2 contains a histadyl tRNA synthetase-like domain that is thought to have the ability to recognize many types of uncharged tRNA, it logically follows that GCN2 may be able to sense both cytosolic and mitochondrial uncharged tRNA [16, 19, 112–115]. Its activation leads to the translation of ATF4 and likely similar downstream responses no matter the type of uncharged tRNA that initiated this cascade, supporting what we found from testing various tRNA synthetase inhibitors that inhibit both cytosolic and mitochondrial tRNA synthetases. To that end, we found that protein synthesis is decreased and proteasomal activity is increased in wild type cells in comparison to their ATF4 KO counterparts. While our current model is that mupirocin, REP3132, and REP8839 act only as inhibitors of their mitochondrial tRNA synthetases respectively, we have not ruled out the possibility that these compounds act on the cytosolic enzymes as well, or even on other previously undiscovered targets.

In order to further assess these drugs’ potential for therapeutic uses, researchers must ensure that the dosing of their organisms leads to the high upregulation of ATF4 or its orthologs. Of note, only certain ranges of concentrations of these drugs greatly increase lifespan in yeast and worms, dependent on their ATF4 orthologs [27]. Under-dosing may have little to no effect, and over-dosing may have a detrimental effect, aligning with other studies (and further evidenced here in Supplemental Fig. 1A) [116].

ATF4 and its orthologs are also known to impact protein synthesis [91, 95, 96, 117]. The results presented here show ATF4 can decrease protein synthesis in response to doses of some inhibitors, but it alone is not responsible for the reduction of protein synthesis in mupirocin, halofuginone, LysRS-IN-2, REP3123, and REP 8839 treated cells. Previous work outlined that borrelidin decreases protein synthesis in both wild type and *gcn4Δ* yeast; however, replicative lifespan is only increased in the wild type strains [27]. This, along with these data here, indicate that lowered translation is not the sole contributor to lifespan increase and that other protein degradation processes regulated by Gcn4/ATF4, such as autophagy or the UPS, may play a necessary role, warranting further investigation. However, these data uncouple elevated proteasome activity and reduced protein synthesis.

Although this manuscript outlines the argument that ATF4 is beneficial in many contexts of aging, there are contexts in which ATF4 plays a negative role [67, 118, 119]. For instance, recent work by Miller et al. has shown that ATF4 KO mice have maintained muscle mass with age [119]. This may be due to the fact that those mice developed without *Atf4*, suggesting that development without *Atf4* can result in alternative pathways regulating the same phenomenon. Another perspective may be that since the data presented here associates *Atf4* with protein degradation mechanisms, perhaps the loss of *Atf4* results in impaired protein degradation capacity and thus decreased atrophy. This could explain why the maintenance of muscle mass is improved in the Miller et al. ATF4 KO mouse model. Further, ATF4 is considered a cancer target as it has been found to be pro-oncogenic; when upregulated, ATF4 can give cancerous cells the ability to live with limited nutrient delivery, potentially due to its positive relationship with protein degradation mechanisms such as autophagy [52, 53, 120–124].

Conceptually, many of the processes found to be enriched in our RNASeq results may have evolved to be influenced by ATF4 in the event the ISR was activated by amino acid deprivation (i.e. the accumulation of uncharged tRNA) to increase amino acid availability and decrease amino acid usage [16, 20, 113, 125]. Similarly, since the integrated stress response is also activated by endoplasmic reticulum stress and the presence of double-stranded RNA, it follows that ATF4 would logically impact genes that would have the ability to respond these activators of the ISR as well [16]. The RNASeq data here illustrates that even though we increased ATF4 translation through GCN2, we found many differentially expressed genes tied to the biological process categories unfolded protein response, viral presence response, and endoplasmic reticulum stress (Fig. 3). These data further outline the roles ATF4 plays in response to ISR activation, consistent with previous findings, as a multifaceted transcription factor [50–52].

Inducing processes that activate pathways known to promote protein turnover has great interest in the development of therapeutics for diseases characterized by protein aggregation, including important diseases of aging such as Huntington’s and Alzheimer’s diseases [105]. The results presented here showing that tRNA synthetase inhibitors greatly upregulate proteasomal degradation as well as autophagy open the door for further exploration of tRNA synthetase inhibitors as potential treatments for these very important diseases of aging.

### Supplementary Information

Below is the link to the electronic supplementary material.

Supplementary file1 (DOCX 25842 KB)

Supplementary file2 (XLSX 2.29 MB)

## Data Availability

All genomic data described in this study are available from the Gene Expression Omnibus (GEO) with accession number #GSE217634.
