# Supplementary material for: Induction of proteasomal activity in mammalian cells by lifespan-extending tRNA synthetase inhibitors
Source: GeroScience. 2023 Sep 25;46(2):1755–73. doi: 10.1007/s11357-023-00938-8 (PMC10828360; doi:10.1007/s11357-023-00938-8)
Supplement: Supplementary file 1 — Supplementary file1 (DOCX 25842 KB) [file 11357_2023_938_MOESM1_ESM.docx]

**Supplementary Figure 1**

**

**

**Supplementary Figure 2**

**
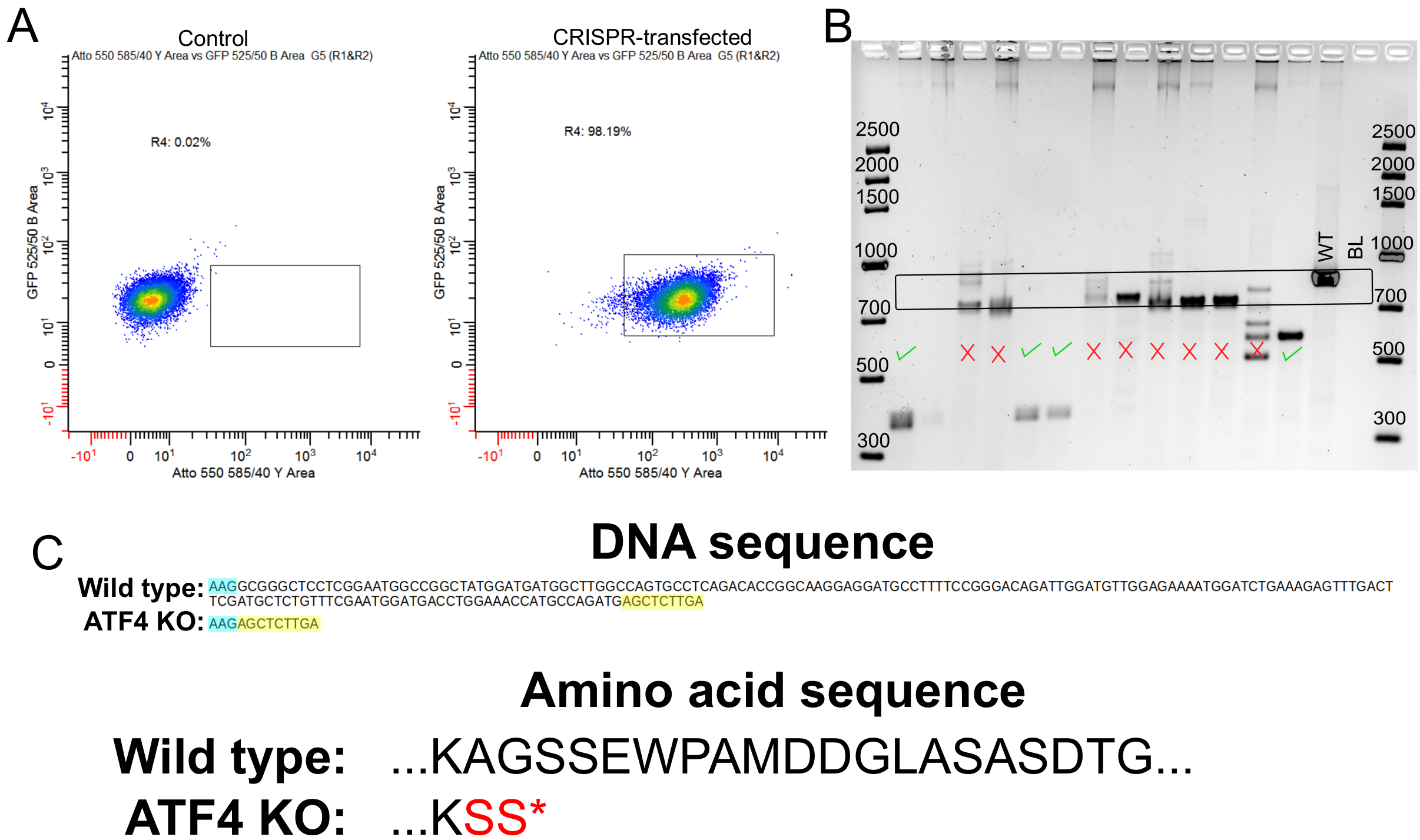
**

**Supplementary Figure 3**

**
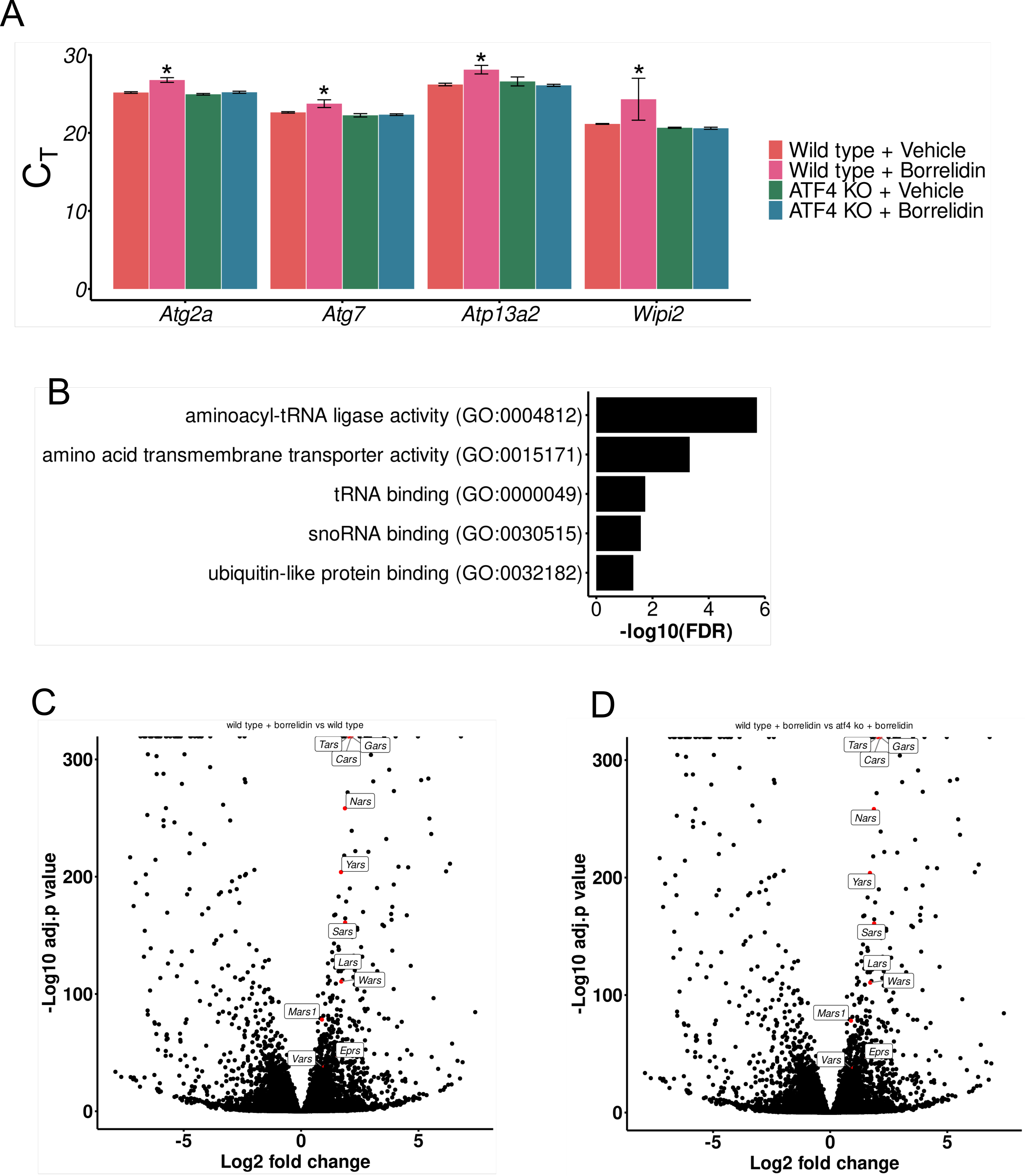
**

**Supplementary Figure 4**

**
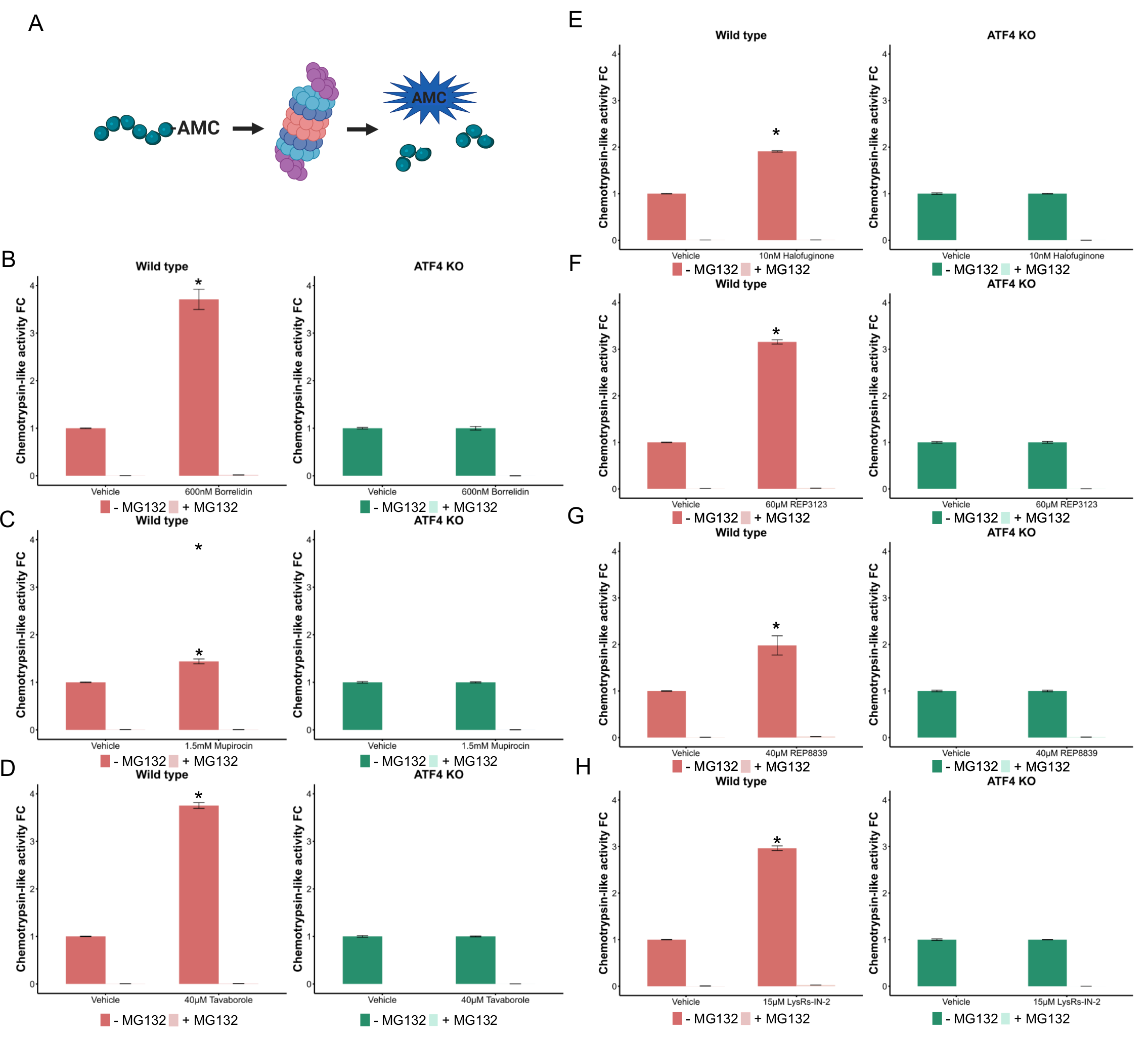
**

**Supplementary Figure 5**
